# Automatic segmentation of trabecular and cortical compartments in HR-pQCT images using an embedding-predicting U-Net and morphological post-processing

**DOI:** 10.1038/s41598-022-27350-0

**Published:** 2023-01-05

**Authors:** Nathan J. Neeteson, Bryce A. Besler, Danielle E. Whittier, Steven K. Boyd

**Affiliations:** McCaig Institute for Bone and Joint Health and Department of Radiology, University of Calgary, Calgary, AB T2N 1N4 Canada

**Keywords:** Imaging, Bone

## Abstract

High-resolution peripheral quantitative computed tomography (HR-pQCT) is an emerging in vivo imaging modality for quantification of bone microarchitecture. However, extraction of quantitative microarchitectural parameters from HR-pQCT images requires an accurate segmentation of the image. The current standard protocol using semi-automated contouring for HR-pQCT image segmentation is laborious, introduces inter-operator biases into research data, and poses a barrier to streamlined clinical implementation. In this work, we propose and validate a fully automated algorithm for segmentation of HR-pQCT radius and tibia images. A multi-slice 2D U-Net produces initial segmentation predictions, which are post-processed via a sequence of traditional morphological image filters. The U-Net was trained on a large dataset containing 1822 images from 896 unique participants. Predicted segmentations were compared to reference segmentations on a disjoint dataset containing 386 images from 190 unique participants, and 156 pairs of repeated images were used to compare the precision of the novel and current protocols. The agreement of morphological parameters obtained using the predicted segmentation relative to the reference standard was excellent (R^2^ between 0.938 and > 0.999). Precision was significantly improved for several outputs, most notably cortical porosity. This novel and robust algorithm for automated segmentation will increase the feasibility of using HR-pQCT in research and clinical settings.

## Introduction

High-resolution peripheral quantitative computed tomography (HR-pQCT) is an in vivo medical imaging tool that provides an isotropic voxel size of 60.7 µm, allowing for precise and direct quantification of cortical and trabecular microarchitectural parameters via morphometric analysis^1^. HR-pQCT is an emerging technology and has the potential to transition from a being research-only tool to become the future of advanced clinical bone densitometry. In combination with micro-finite element modelling (µFEM)^2,3^, HR-pQCT has provided insight into how trabecular microarchitecture at the distal radius and tibia is affected by a wide variety of factors, including age, sex, physical activity, disease, and nutrition^4–9^. HR-pQCT shows particular promise for fracture screening, as several studies have shown that bone morphology and phenotypes at peripheral skeletal sites are independently and significantly predictive of fracture risk^10–12^.

**Figure 1:**
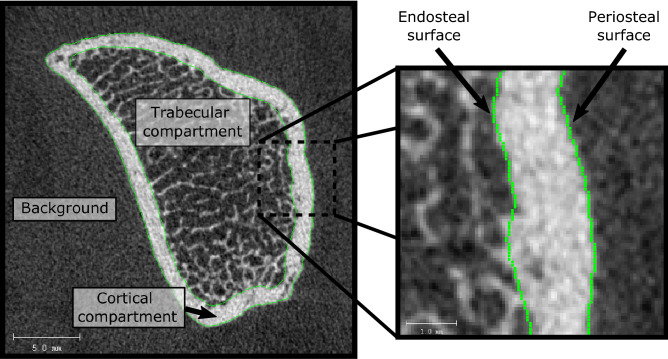
Axial 2D slice of HR-pQCT radius image cropped close to the bone. The cortical, trabecular, and background compartments are labelled, as are the periosteal and endosteal surface contours. The periosteal surface is the interface between the background and the cortical region, while the endosteal surface is the interface between the cortical and trabecular regions.

A limitation of HR-pQCT is that standard quantitative morphometric analysis of radius and tibia images requires an accurate semantic segmentation of the trabecular and cortical bone compartments (Fig. 1). The current gold-standard segmentation protocol requires human operators to manually inspect and correct automatically generated segmentations^13^. Manual correction is time-consuming and introduces potential for inter- and intra-operator biases, particularly with untrained or inexperienced operators. This has been recently demonstrated in a study performed in our lab that found systematic biases exceeding the least significant change (LSC) when uncorrected segmentations were used, and a significant influence of operator experience level on precision errors even with corrected segmentations^14^. With the current segmentation protocol, the skill and experience level of the operator has a large effect on both the accuracy and precision of the analysis, and thus on the reliability of study outcomes. The requirement for manual inspection and correction, and the potential biases these processes incur, poses a significant barrier to wider adoption of this technology in both clinical and research settings.

Development of robust segmentation protocols for distal radius and tibia images has been an active area of research since HR-pQCT became available. Originally, segmentations were produced manually, slice-by-slice, assisted by the Snakes edge-finding algorithm^15,16^. This method was later supplanted in common practice by an automatic dual-thresholding algorithm^17^, and a modified version of this method remains the current gold standard for generating preliminary segmentations^18^. However, the dual-threshold technique was developed using assumptions based on typical bone characteristics. It can fail to distinguish cortical from trabecular bone in atypical cases, such as professional athletes (high bone quality) and osteoporotic patients (low bone quality). Consequently, the segmentations must be manually inspected for deviations and corrected^13^, and the specific types and prevalence of errors that must be corrected have been documented in prior studies^14^. There have been several automatic segmentation approaches developed recently, including the combination of image texture analysis with machine learning^19^, multiple dual-active contours approaches^20,21^, and a deep learning-based computer vision approach^22^. However, there has yet to be a segmentation technique developed for standard HR-pQCT images at distal sites that sufficiently matches or exceeds performance of the current semi-automated gold standard.

Convolutional neural networks (CNNs) were first introduced nearly three decades ago for computer vision^23^. Since the breakthrough development of AlexNet in 2012^24^, development and application of CNNs has greatly accelerated for several tasks and domains^25–27^. In particular, the development of the fully connected network (FCN)^28^ and the U-Net^29,30^ were critical developments for the advancement of biomedical image segmentation. U-Nets have been shown to accurately automate semantic segmentation across a broad range of biomedical imaging modalities and applications^31^. Recently, standard 2D and 3D U-Nets have been successfully applied to the task of segmenting HR-pQCT hand images^22^, although the aim was whole bone segmentation for estimation of total volumetric bone mineral density, rather than defining cortical and trabecular compartments separately for full morphometric analysis of bone microarchitecture.

The objective of this study is to develop and evaluate a fully automated, end-to-end algorithm to replace the current standard semi-automated method for segmenting HR-pQCT distal tibia and radius images. The proposed protocol combines a modified U-Net segmentation model with a morphological post-processing algorithm that is specifically designed for the task of segmenting HR-pQCT radius and tibia images in preparation of quantitative morphological analysis. Robust automation of HR-pQCT image analysis will incent wider adoption of HR-pQCT technology in the bone densitometry research community and make feasible clinical adoption of HR-pQCT.

## Results

After stratified splitting of the dataset, there were 896, 190, and 190 unique participants in the *training*, *validation*, and *test* subsets, respectively. There were 326 men and 570 women in the *training* dataset, 67 men and 123 women in the *validation* dataset, and 64 men and 126 women in the *test* dataset. The *training* dataset contained 885 radii and 937 tibiae, the *validation* dataset contained 187 radii and 203 tibiae, and the *test* dataset contained 185 radii and 201 tibiae.

Using the trained segmentation model and post-processing algorithm, the mean time to produce a post-processed 3D predicted segmentation for a full radius or tibia image in the *test* subset was 140 s (SD 56 s) on a research computing cluster node with an NVIDIA Tesla V100 and an Intel Xeon Gold 6148. Inference and post-processing times scale with the size of the image, which depends on the size of the radius or tibia and how close the image has been cropped to the bone.

### Held-out test subset

**Figure 2:**
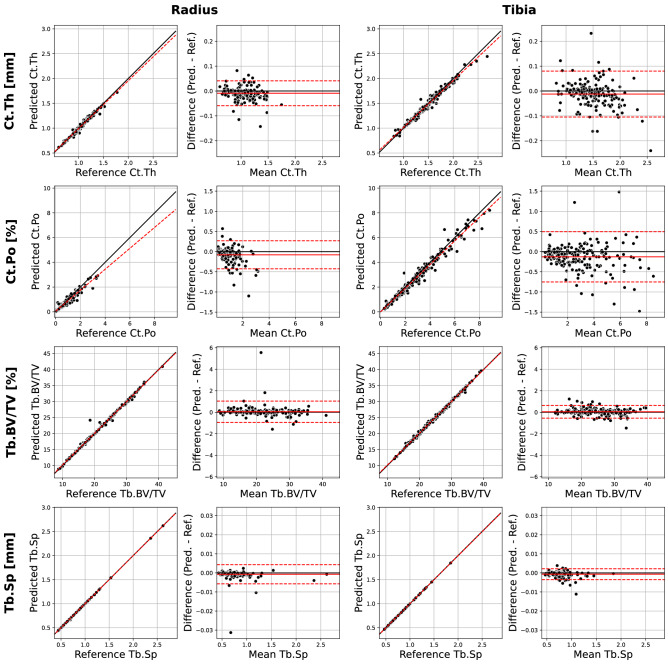
Linear regression and Bland–Altman plots for select parameters comparing results of morphometric analysis on the held-out test set using predicted and reference segmentations. Linear regression: the dashed black line is the line of unity, while the dashed red line is the linear fit between the predicted and reference outputs. Bland–Altman: the solid black line is the line of zero error, the dashed red line indicates the mean bias error, and the dashed black lines indicate the 95% limits of agreement (n = 185 for radius; n = 201 for tibia).

The comparative analysis of predicted and reference segmentations on the *test* subset are shown in Tables 1 and 2 and in Fig. 2. Dice similarity coefficient (DSC) and Jaccard similarity coefficient (JSC) are reported separately for each compartment while Hausdorff distance and average symmetric surface distance (ASSD) are reported separately for each surface. For both the radius and the tibia, the mean DSC of the predicted cortical and trabecular segmentations are ≥ 0.97 and ≥ 0.99, indicating extremely close segmentation overlap across the *test* subset. The mean ASSD is no greater than 0.08 mm (less than one and a half times the width of a 0.0607 mm voxel) for both surfaces in both scan sites in the *test* subset and the mean Hausdorff distances are less than 0.91 mm (approximately 15 voxels) for both surfaces in both scan sites across the *test* subset.

**Table 1:** Results of direct comparison of predicted and reference masks on held-out test dataset.

		Mean (SD)	(Min, Max)
**Radius (n = 185)**			
Cortical Mask	DSC	0.98 (0.02)	(0.90, 0.99)
	JSC	0.94 (0.03)	(0.82, 0.98)
Trabecular Mask	DSC	0.99 (< 0.01)	(0.97, > 0.99)
	JSC	0.99 (0.01)	(0.94, 0.99)
Endosteal Surface	Hausdorff (mm)	0.6 (0.3)	(0.2, 1.9)
	ASSD (mm)	0.05 (0.02)	(0.02, 0.18)
Periosteal Surface	Hausdorff (mm)	0.3 (0.2)	(0.1, 1.0)
	ASSD (mm)	0.02 (0.01)	(0.01, 0.06)
**Tibia (n = 201)**			
Cortical Mask	DSC	0.97 (0.02)	(0.90, 0.98)
	JSC	0.93 (0.03)	(0.81, 0.97)
Trabecular Mask	DSC	0.99 (< 0.01)	(0.98, > 0.99)
	JSC	0.99 (0.01)	(0.96, > 0.99)
Endosteal Surface	Hausdorff (mm)	0.9 (0.4)	(0.3, 3.5)
	ASSD (mm)	0.08 (0.03)	(0.04, 0.21)
Periosteal Surface	Hausdorff (mm)	0.4 (0.3)	(0.2, 2.7)
	ASSD (mm)	0.02 (0.01)	(0.01, 0.09)

*DSC* Dice similarity coefficient, *JSC* Jaccard similarity coefficient, *Hausdorff* maximum symmetric surface distance, *ASSD* average symmetric surface distance.

**Table 2:** Results of linear regression and Bland–Altman analysis on the held-out *test* dataset, comparing the predicted morphometric outputs, obtained using predicted segmentations, and the reference morphometric outputs, obtained using reference segmentations.

	Bland–Altman		Linear Regression		
	Mean Error (95% LOA^a^)		Slope (95% C.I.^b^)	Intercept (95% C.I.^b^)	R^2^
**Radius (n = 185)**					
Tt.BMD	mg HA/cm^3^	0.6 (− 3.3, 4.6)	0.995 (0.991, 1.000)	2.0 (0.6, 3.4)	0.999
Ct.BMD	mg HA/cm^3^	− 0.9 (− 21.4, 19.7)	1.013 (0.990, 1.037)	− 12.7 (− 33.6, 8.3)	0.975
Tb.BMD	mg HA/cm^3^	0.2 (− 6.2, 6.6)	0.990 (0.980, 1.000)	1.8 (0.1, 3.4)	0.995
Ct.Th	mm	− 0.01 (− 0.06, 0.04)	0.963 (0.946, 0.980)	0.030 (0.012, 0.048)	0.986
Ct.Po	%	− 0.08 (− 0.43, 0.27)	0.847 (0.816, 0.878)	0.050 (0.016, 0.083)	0.938
Tb.BV/TV	%	0.047 (− 0.943, 1.037)	0.991 (0.980, 1.001)	0.253 (0.004, 0.502)	0.994
Tb.N	mm^−1^	0.000 (− 0.004, 0.004)	1.000 (0.999, 1.001)	0.000 (− 0.001,0.002)	> 0.999
Tb.Th	mm	0.000 (− 0.007, 0.007)	0.964 (0.939, 0.988)	0.009 (0.003, 0.014)	0.970
Tb.Sp	mm	− 0.001 (− 0.006, 0.004)	0.999 (0.997, 1.000)	0.000 (− 0.001, 0.001)	> 0.999
Tt.Ar	mm^2^	0.6 (− 0.8, 1.9)	1.001 (1.000, 1.003)	0.2 (− 0.2, 0.7)	> 0.999
Ct.Ar	mm^2^	0.1 (− 3.1, 3.2)	0.957 (0.944, 0.971)	2.7 (1.9, 3.6)	0.991
Tb.Ar	mm^2^	0.5 (− 2.6, 3.5)	1.004 (1.000, 1.008)	− 0.5 (− 1.3, 0.4)	0.999
**Tibia (n = 201)**					
Tt.BMD	mg HA/cm^3^	0.1 (− 2.5, 0.4)	0.999 (0.999, 1.000)	0.3 (0.2, 0.4)	> 0.999
Ct.BMD	mgHA/cm^3^	3.8 (− 14.3, 21.9)	0.975 (0.962, 0.988)	25.2 (14.4, 36.1)	0.991
Tb.BMD	mgHA/cm^3^	0.3 (− 4.4, 4.9)	0.990 (0.982, 0.998)	1.9 (0.6, 3.2)^†^	0.997
Ct.Th	mm	− 0.01 (− 0.11, 0.08)	0.944 (0.924, 0.963)	0.072 (0.042, 0.101)	0.979
Ct.Po	%	− 0.13 (− 0.75, 0.49)	0.957 (0.935, 0.979)	− 0.008 (− 0.083, 0.068)	0.973
Tb.BV/TV	%	0.044 (− 0.547, 0.634)	0.991 (0.984, 0.998)	0.263 (0.085, 0.441)	0.997
Tb.N	mm^−1^	0.000 (− 0.004, 0.005)	1.000 (0.998, 1.001)	0.001 (− 0.001, 0.003)	> 0.999
Tb.Th	mm	0.000 (− 0.006, 0.007)	0.985 (0.969, 1.001)	0.004 (> 0.000, 0.008)	0.987
Tb.Sp	mm	− 0.001 (− 0.004, 0.002)	0.998 (0.997, 0.999)	0.001 (> 0.000, 0.002)	> 0.999
Tt.Ar	mm^2^	0.3 (− 0.9, 1.4)	1.000 (1.000, 1.001)	− 0.1 (− 0.5, 0.3)	> 0.999
Ct.Ar	mm^2^	− 1.2 (− 8.7, 6.3)	0.956 (0.940, 0.972)	4.6 (2.5, 6.8)	0.986
Tb.Ar	mm^2^	1.3 (− 6.1, 8.8)	1.002 (0.998, 1.006)	0.0 (− 2.3, 2.5)	0.999

^a^95% limits of agreement (LOA) are the mean error plus or minus 1.96 times the standard deviation of the errors.

^b^95% confidence interval (C.I.) are the estimated slope or intercept plus or minus 1.96 times the estimated standard error of the estimate, as reported by statsmodels’ ordinary least squares linear regressor after being fit to the data.

Figure 2 compares results of a quantitative morphometric analysis via linear regression and Bland–Altman plots for cortical thickness (Ct.Th), cortical porosity (Ct.Po), trabecular bone volume fraction (Tb.BV/TV), and trabecular separation (Tb.Sp), while Table 2 tabulates summary statistics of Bland–Altman and linear regression analysis for all standard analysis outputs, separated by scan site, each evaluated using the full *test* subset. All parameters at both scan sites have limits of agreement that overlap with zero mean bias error. Nearly all parameters have coefficients of determination that are ≥ 0.97, where the lone exception is Ct.Po in the radius (R^2^ = 0.938). There were no substantial differences (95% confidence interval for slope estimate contained the unit slope) between the fitted slopes and the null hypothesis unit slope for 13 of the total 24 parameters. While there are substantial differences between the linear fit and the null zero-intercept, unit-slope linear model for 11 parameters, all but one of these deviating parameters have estimated slopes between 0.944 and 1.004. The outlying parameter is again Ct.Po in the radius, with a fitted slope of 0.847 ± 0.031. This discrepancy is reflected visually in the second row, left column of Fig. 2, where both the linear correlation and Bland–Altman plots for Ct.Po in the radius shows several extreme over-estimates of predicted Ct.Po where the mean value is low (< 1%) and several extreme under-estimates of predicted Ct.Po where the mean value is high (> 2%).

The linear correlation and Bland–Altman sub-analyses on the “low cortical thickness” and “high cortical porosity” sub-groups are tabulated in Tables S.1 and S.2 in the Supplementary Data. Referring to Table S.1 for the “low cortical thickness” sub-group: Coefficients of determination were > 0.93 for all parameters, where the two lowest values were for Ct.Th in the radius and tibia. Zero mean bias was observed for all parameters, and the quality of the linear fits were not substantially different from those in the main group (Table 2). Referring to Table S.2 for the “high cortical porosity” sub-group: Coefficients of determination were > 0.94 for all but two parameters, these being Ct.Po in the radius and tibia, where the coefficients of determination were 0.810 and 0.816, respectively. Zero mean bias was observed for all parameters. The quality of the linear fits were not substantially different from those in the main group (Table 2) for all parameters except Ct.Po in the radius and tibia, where the fitted slopes were 0.813 ± 0.116 and 0.893 ± 0.101, respectively. This indicates both a tendency to under-estimate Ct.Po and a greater variance in predicted Ct.Po values relative to reference values, for images with larger reference Ct.Po values.

### Sample visual results

**Figure 3:**
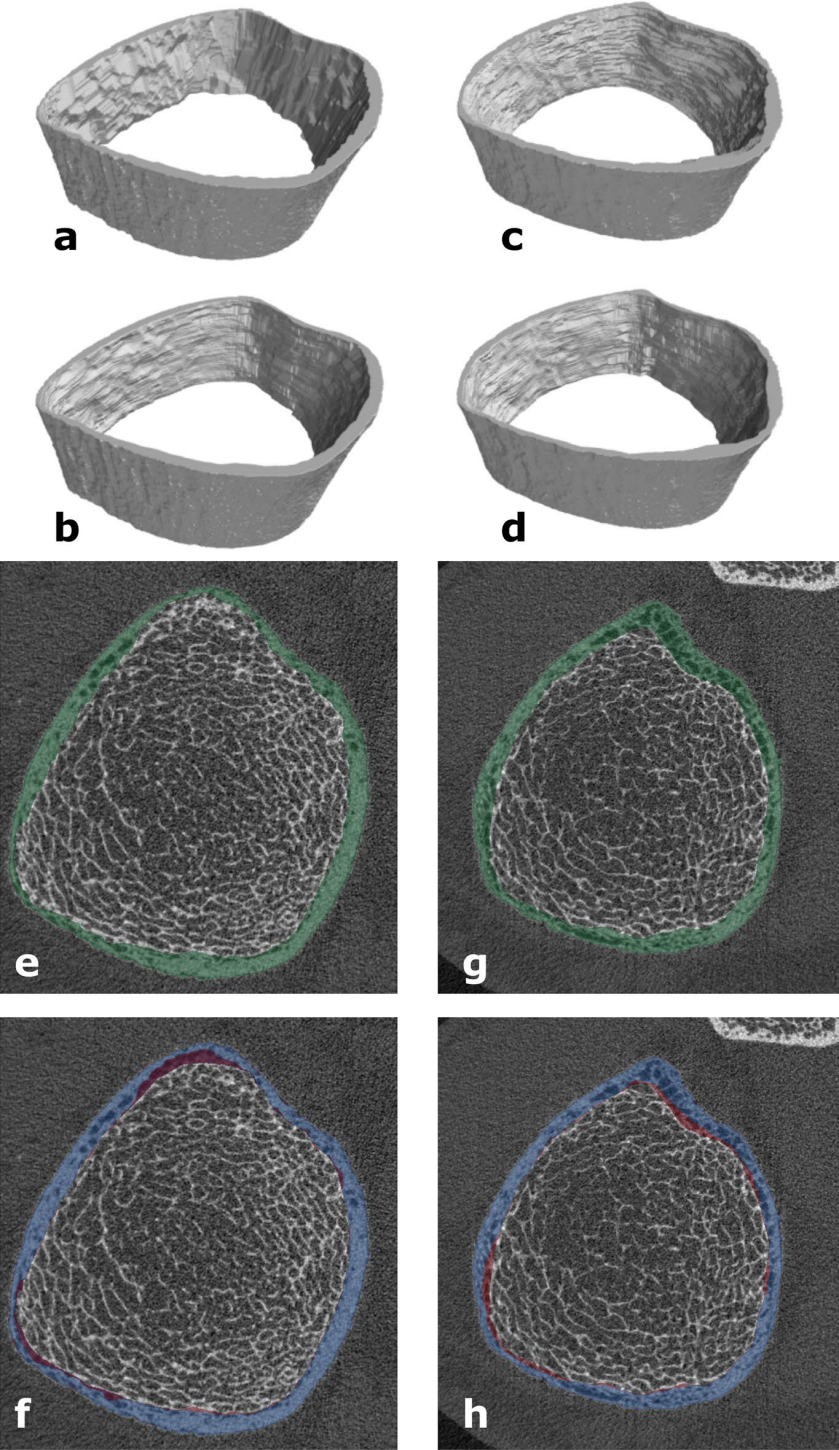
Sample visualization of the two images in the *test* dataset with the most extreme over- and under-estimates of Ct.Po (predicted value less reference value). Both are distal tibia images. The largest over-estimate of Ct.Po was + 1.48% (left column: **a**,**b**,**e**,**f**) while the largest under-estimate of Ct.Po was -1.48% (right column: **c**,**d**,**g**,**h**). (**a–d**) 3D surface renderings are shown of reference (**a**,**c**) and predicted (**b**,**d**) cortical compartment segmentations. (**e–h**) The image is shown in all panes in grayscale, overlaid with colored masks corresponding to the reference (**e**,**g**—green), the prediction (**f**,**h—**blue), and the voxels for which there is disagreement (**f**,**h—**red).

While the discrepancy in Ct.Po between the predicted and reference segmentations is less than 0.5% for > 93% of the images in the *test* subset, we visually explore specific cases of extreme disagreement to gain insight into how they arise. Accordingly, Fig. 3 shows sample visualizations of predicted and reference masks, and disagreement, for three axial slices from the two images in the *test* subset with the largest over- and under-estimates in Ct.Po. Disagreements arise primarily along the endosteal surface in regions where, when looking at a single slice, it is ambiguous whether a specific structure corresponds to porous cortical zones or thickened near-endosteal trabeculae. Figure 3 also includes volumetric surface renderings of the reference and predicted cortical compartment for these two images, to qualitatively demonstrate the overall similarity in shape and surface smoothness.

### Held-out precision subset

Table 3 shows the root-mean-square percentage coefficient of variance (RMS %CV) and least significant change (LSC) values, in parameter-specific units, for all standard quantitative analysis outputs, evaluated with the full *precision* subset. RMS %CV and LSC are reported separately for scan site and for each of the current semi-automated gold standard and proposed segmentation protocols. Root-mean-square standard deviation (RMS SD) values are provided in Table S.3 in the Supplementary Data. D’Agostino and Pearson’s test for normality indicated that the individual standard deviations were not normally distributed for most parameters. Therefore, for consistency, the significance of the differences in the coefficients of variation were assessed using independent Wilcoxon signed-rank tests for all parameters.

**Table 3:** Root-mean-square percentage coefficient of variation (RMS %CV) and least significant change (LSC) compared between the proposed algorithm (U-Net) and the current semi-automated gold standard (Standard) segmentation protocols on the held-out *precision* dataset.

		RMS %CV		LSC		Wilcoxon *p* Value
		U-Net^a^	Standard^b^	U-Net	Standard	
**Radius (n = 71)**						
Tt.BMD	mg HA/cm^3^	0.29	0.70	2.51	5.60	0.796
Ct.BMD	mg HA/cm^3^	0.27	0.79	6.67	19.12	0.010^c^
Tb.BMD	mg HA/cm^3^	0.51	0.61	1.87	2.37	0.055
Ct.Th	mm	0.69	0.80	0.022	0.025	0.781
Ct.Po	%	7.92	9.55	0.23	0.25	0.001^c^
Tb.BV/TV	%	1.00	1.06	0.51	0.54	0.030^c^
Tb.N	mm^−1^	1.40	1.41	0.057	0.057	0.030^c^
Tb.Th	mm	0.63	0.72	0.004	0.005	0.005^c^
Tb.Sp	mm	1.06	1.07	0.021	0.022	0.036^c^
Tt.Ar	mm^2^	0.71	0.77	6.20	5.01	0.000^c^
Ct.Ar	mm^2^	0.76	1.17	1.46	1.78	0.000^c^
Tb.Ar	mm^2^	0.79	1.27	5.35	6.29	0.284
**Tibia (n = 85)**						
Tt.BMD	mg HA/cm^3^	0.47	0.48	3.72	3.74	0.639
Ct.BMD	mg HA/cm^3^	0.36	0.45	8.37	10.47	0.000^c^
Tb.BMD	mg HA/cm^3^	0.61	0.69	2.79	3.06	0.016^c^
Ct.Th	mm	0.61	0.74	0.026	0.032	0.333
Ct.Po	%	9.07	11.67	0.76	0.82	0.045^c^
Tb.BV/TV	%	0.83	0.82	0.50	0.52	0.560
Tb.N	mm^−1^	2.97	2.98	0.11	0.11	0.246
Tb.Th	mm	0.56	0.67	0.004	0.005	0.460
Tb.Sp	mm	2.21	2.21	0.045	0.045	0.497
Tt.Ar	mm^2^	0.27	0.13	5.63	2.74	0.000^d^
Ct.Ar	mm^2^	0.37	0.66	1.41	2.42	0.991
Tb.Ar	mm^2^	0.29	0.16	4.92	2.68	0.000^d^

^a^U-Net: automated segmentation algorithm using a U-Net and morphological post-processing.

^b^Standard: Current standard semi-automated segmentation protocol.

^c^Wilcoxon signed-rank test indicates significantly lower individual standard deviations with automated segmentation algorithm as compared to the standard semi-automated protocol.

^d^Wilcoxon signed-rank test indicates significantly higher individual standard deviations with automated segmentation algorithm as compared to the standard semi-automated protocol.

The novel automated segmentation algorithm produced equivalent or significantly better precision for all morphometric parameters except for total area (Tt.Ar) and trabecular area (Tb.Ar) in the tibia (Tt.Ar: RMS CV% was 0.27% and 0.13% for the novel and standard protocols, respectively; Tb.Ar: RMS CV% was 0.29% and 0.16% for the novel and standard protocols, respectively). For most of these parameters, the magnitudes of the significant differences were small, ranging between -0.13% and 0.52% (negative indicating worse precision and positive indicating improved precision). The exception is the Ct.Po in both the radius and tibia. In the radius, the RMS %CV of the novel and standard protocols were 7.92% and 9.55%, respectively. In the tibia, the RMS %CV of the novel and standard protocols were 9.07% and 11.67%, respectively. These statistically significant differences indicate an absolute reduction in variation, and thus improvement in precision, of 1.63% and 2.60% in the radius and tibia, respectively, for cortical porosity measurements with the novel, automated segmentation protocol.

Precision analysis results for “low cortical thickness” and “high cortical porosity” sub-groups are tabulated in Table S.4 in the Supplementary Data. With the “low cortical thickness” sub-group, the proposed algorithm produces outputs with equivalent or better precision for all morphometric parameters with the exception of Tt.Ar in the tibia. Precision was statistically significantly better with the proposed algorithm than with the standard protocol for Ct.Th in the radius, Ct.Po in the radius, and Tb.N in the radius and tibia. RMS %CV values for Ct.Th and Ct.Po in the tibia were lower with our algorithm than with the standard protocol, but the differences were not statistically significant.

With the “high cortical porosity” sub-group, the proposed segmentation algorithm produces outputs with equivalent or better precision for all morphometric parameters. Precision was statistically significantly better with our algorithm than with the standard protocol for Ct.BMD in the radius, Tb.N in the tibia, and Ct.Ar in the tibia. RMS %CV values for Ct.Th in the tibia and radius and Ct.Po in the radius were lower with the proposed algorithm than with the standard protocol, while the RMS %CV values for Ct.Po in the tibia were higher with the proposed algorithm than with the standard protocol; however, none of these differences were statistically significant.

## Discussion

This study proposes a segmentation algorithm that utilizes a 2D U-Net applied to stacks of five axial slices at a time to obtain preliminary 3D segmentations. These segmentations are then morphologically post-processed to ensure physiological validity. The combination of machine learning with traditional image processing is a truly novel approach for HR-pQCT image segmentation. The fully automated algorithm was able to segment trabecular and cortical compartments at least ten times faster than the current gold-standard protocol (2–3 min versus up to 30 min), with superior precision, while requiring no human intervention or oversight. The predicted segmentations were found to be accurate when compared to reference segmentations based on both traditional medical image segmentation metrics and by comparing predicted and reference morphological analysis outputs, using a large *test* dataset comprised of 386 total images from 190 participants. Predicted segmentations were also found to be as precise or more precise when compared to reference segmentations from 156 same-day, repeat-scan image pairs from 90 participants.

To the authors’ knowledge, this is the first study to present a fully automated algorithm for segmentation of HR-pQCT radius and tibia images that requires no manual correction and demonstrates greater or equal precision when compared to the current standard segmentation protocol. While a previous study successfully explored the use of 2D and 3D U-Nets for segmentation of HR-pQCT hand images^22^, the U-Nets in that study were designed to extract the entire bone for estimation of Tt.BMD—a simpler task than semantic segmentation of the cortical and trabecular compartments for a complete morphometric analysis of distal bone microarchitecture. There have been several recent attempts to automate segmentation of radius and/or tibia HR-pQCT images using dual active contours and texture analysis combined with machine learning. However, none of these techniques have supplanted the current semi-automated protocol in standard practice, due to varying limitations. These techniques were either exclusively focused on obtaining only the periosteal contour^21^, did not quantify the accuracy of subsequent morphometric analysis^20^, or had insufficient agreement between reference and generated segmentations (DSC = 0.90 ± 0.05). Further, no prior work presenting a new automated pipeline for HR-pQCT segmentation has quantified the precision of the proposed protocol.

The proposed algorithm requires no human intervention or correction, in contrast to the current standard protocol, where correction of individual images may occupy a human operator for as much as 30 min for challenging images^14^. With the proposed algorithm, standard analysis-compatible cortical and trabecular masks are ready in approximately 2 min and 20 s. Further, processing can be batched while researchers attend to other tasks or parallelized on a computing cluster if a large dataset needs to be processed quickly. However, the elimination of human intervention would not only save time. Automation prevents inter-operator error since the algorithm is deterministic and will always create the same segmentation from the same input image. Inter-operator error can materially impact precision and LSC in both multi-center and longitudinal studies, where it can often be infeasible to have a single expert operator correct all segmentations consistently.

While the predicted segmentations are highly accurate for > 93% of images, disagreements between reference and predicted segmentations can still occur; however, we have shown visually that the most extreme of these disagreements occur in places where a human operator may experience the same challenge in defining compartments. This observation is further reinforced by the tendency for the Hausdorff distance and ASSD to be much lower at the periosteal surface than the endosteal surface, by at least a factor of two. The correct label or classification for voxels in these low-density regions can be unclear even for the human observer, as the structures could be interpreted as either porous cortical zones or thickened trabecular zones. The semi-automated segmentation correction protocol does not provide rigorous criteria for how to differentiate between these cases, so human operators must make inherently subjective real-time decisions when correcting segmentations. Whether the proposed algorithm or standard semi-automated protocol more often achieve the objectively correct endosteal surface, or whether such a thing even exists, is unclear. However, it is apparent from analysis of the *precision* data that this surface is selected with greater consistency and reproducibility with the proposed algorithm than it is with the semi-automated protocol. This is evinced by the statistically significant decreases of RMS %CV for Ct.Po from 9.55 to 7.92% in the radius and from 11.67 to 9.07% in the tibia (Table 3). The inherent difficulty of correctly placing the endosteal surface in the presence of low-density cortical bone also explains the relatively worse linear fit for cortical porosity in the radius and the larger scatter observed in the linear regression plots for this parameter in both scan sites in the analysis of the *test* data.

There are important limitations in this study that should be considered. First, all the image data used for training and evaluation were produced using the same scanning parameters at two specific standard distal scan sites. The distal radius and tibia are the standard HR-pQCT scan sites, and the vast majority of the usage of HR-pQCT for basic and translational research focuses on these sites^32^. However, there is growing interest in applying HR-pQCT to study in vivo bone microarchitecture at other sites, including the knee^33^ and the elbow^34^. Extension of the proposed algorithm to other scan sites may be achievable using domain adaptation techniques, such as transfer learning^35^, and would undoubtedly require adjustments to the post-processing procedure. It is not currently clear how difficult this adaptation process would be, or if the proposed algorithm would be as successful for segmentation of these alternate scan sites as it has been shown to be for the distal radius and tibia.

Second, all the segmentations used for training and evaluation in this study were produced, or reviewed, by an experienced operator at a single research centre using data from participants from a single region in Canada. Generated segmentations and morphometric outputs were also compared only to reference segmentations created by that same experienced operator, albeit on unseen data. Comparing segmentations generated by the proposed algorithm to segmentations created by additional operators would undoubtedly result in increased variance in the comparison results^14^, but the benefit of a fully automated approach is the consistency of the output, which could be shared across all sites and at all timepoints when this approach is used. At the same time, by training on data with segmentations produced by a single expert operator, specific inferential bias has been encoded into the model that informs how the periosteal and endosteal surfaces are placed. While this will not affect the precision of the resulting segmentations or corresponding analysis outputs, it could influence the accuracy when compared to segmentations generated using standard protocols employed at other research centres. An alternative approach for training and developing this algorithm could be to involve a multi-national, multi-center collaboration that produces a large dataset of images and reference segmentations. This approach would both incorporate input from different centres and include study participants representing different geographic areas and ethnicities. In the interim, potential users who are concerned about the agreement between segmentations generated by the proposed algorithm and the segmentations created by their own operators with the standard protocol could perform comparative studies using new unseen data or, if necessary, retrain the 2D U-Net on their own datasets.

In a fully automated system, it would be beneficial to establish robust error detection. However, to capture errors, one must define a ‘failed segmentation’ in the absence of a reference segmentation to compare to. A simple definition would be a segmentation that either misses large portions of the structures of interest or that erroneously includes portions of the background or secondary bone. We did not observe any failed segmentations of this kind in the combined 1088 images in the *validation*, *test*, and *precision* data subsets; however, this is not conclusive evidence that the algorithm will always succeed. A reasonable protocol using the proposed automated protocol will likely still require a human operator to quickly inspect output segmentations to screen for these types of obvious failures.

Development of an automated error screening algorithm is a subject for future work but could include: monitoring axial slice-to-slice variations in the segmentations, monitoring simple morphological metrics of the segmentations (such as the number of connected components in each compartment in the final segmentation), flagging segmentations where the difference between the raw U-Net output and post-processing output exceeds some threshold, or using unsupervised or supervised machine learning methods for outlier detection^36^ with the morphometric analysis outputs and/or the segmentations directly.

## Methods

### Datasets

All images used in this study were obtained using HR-pQCT (XtremeCT II, Scanco Medical AG, Brütisellen, Switzerland) with the standard in vivo protocol^1,13^, at standard scan sites for the distal radius and tibia^37^. In each image, 168 slices were collected with a nominal isotropic voxel size of 60.7 µm. Images were excluded if the motion score was recorded as a four or higher on a one-to-five scale^38^. All participants provided written informed consent prior to data collection, which was approved by the Conjoint Health Research Ethics Board at the University of Calgary (REB16-1606, REB15-0858), and all methods were carried out in accordance with relevant guidelines and regulations. All images used in this study have corresponding reference segmentations, produced by an expert following the standard semi-automated protocol. There were four distinct sets of data used in this study, referred to as the *training*, *validation*, *test*, and *precision* subsets.

#### Train, validate, test subsets

The *training*, *validation*, and *test* subsets were produced by pooling together all second-generation HR-pQCT images from two cross-sectional studies: a normative study (n = 1236)^8^ and a hip fracture study (n = 108)^12^. Participants in the combined dataset were stratified by study then split into four equally sized groups based on total volumetric bone mineral density (Tt.BMD), obtained using reference segmentations. There are multiple images, and therefore multiple Tt.BMD values, for each participant. The minimum Tt.BMD across all images corresponding to each participant was used for the purposes of stratification. Finally, participants in each stratified group were randomly assigned to the *training*, *validation*, or *test* subsets with 70% of participants assigned to the *training* subset, and 15% to each of the *validation* and *test* subsets. In total, there were 1257 radius and 1343 tibia images from 1278 participants used in the *training*, *validation*, and *test* subsets combined.

The *training* dataset was used to train the U-Net’s internal parameters. The *validation* dataset was used to evaluate the U-Net’s performance after training, to inform selection of U-Net architecture and training hyper-parameters, and to develop the post-processing algorithm. The *test* dataset was set aside immediately following subset splitting to prevent data leakage. It was accessed only once to evaluate the performance of the overall pipeline on unseen data, after satisfactory performance was achieved on the validation dataset.

#### Precision subset

The *precision* dataset is fully disjoint from the *training*, *validation*, and *test* datasets and was derived from two separate previous studies^37,39^. The *precision* dataset served as a second held-out dataset, but with repeated same-day scans of the same sites in the same participants. There were 85 tibia and 71 radius images from 90 participants in the *precision* dataset, with a duplicate, same-day scan with repositioning for each image. In the *precision* dataset there were 46 men and 44 women with a combined mean age of 64 years (SD 8 years). The repeated scans allow for an unbiased, quantitative comparison of the precision error and LSC^40,41^ between the proposed and current standard segmentation protocols.

### Pre-processing

During training, HR-pQCT images are imported and converted from native units to densities in milligrams hydroxyapatite per cubic centimeter (mg HA/cm^3^), and reference cortical and trabecular masks are imported and converted to binary images. A padding transform aligns the image and masks and pads the coronal and sagittal extents to the same multiple of eight voxels. A standardization transform performs a truncated linear mapping on the image densities from the interval [− 400, 1400] mg HA/cm^3^ to the unitless interval [− 1, 1] using the following equations:

1 $$\begin{array}{*{20}c} {\rho^{\prime}\left( {{\text{x}},{\text{y}},{\text{z}}} \right) = \min \left( {\max \left( {\rho \left( {x,y,z} \right), - 400} \right),1400} \right),} \\ \end{array}$$

2 $$\begin{array}{*{20}c} {\overline{\rho }\left( {x,y,z} \right) = \frac{{2\rho^{{{^{\prime}}\left( {x,y,z} \right)}} - 1000}}{1800}, } \\ \end{array}$$

where $\rho$ is the image densities, $\rho ^{\prime}$ is the truncated image densities, and $\overline{\rho }$ is the truncated and rescaled image densities on the interval [− 1, 1], which are then used as inputs to the U-Net.

### Embedding-predicting U-Net

#### Architecture

**Figure 4:**
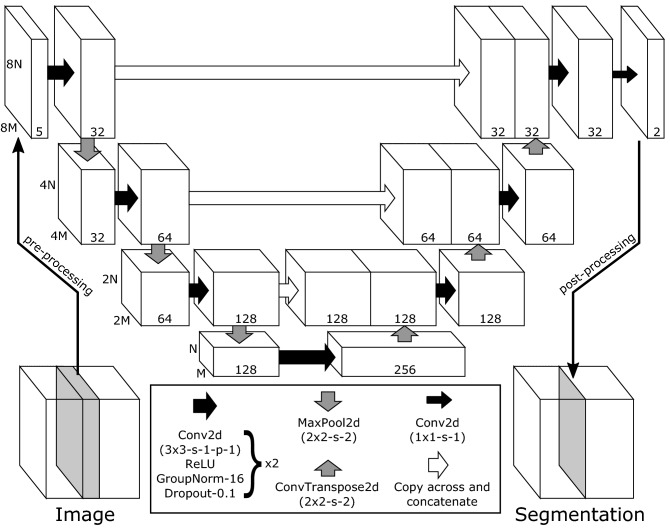
Schematic of modified U-Net architecture. Each box represents a PyTorch tensor containing an input, latent, or output image. All tensors in the same row have the same height and width, but depth (number of channels) varies and is labelled for all tensors. Block arrows represent transitions between tensors and are described in a legend, where ‘s’ refers to stride and ‘p’ refers to padding. Pre-processing refers to padding, scaling, and conversion to PyTorch tensor format, while in this context post-processing refers to converting output surface embeddings to binary masks (cortical and trabecular compartments and background).

The U-Net used in this work, shown schematically in Fig. 4, is based on the original 2D biomedical U-Net architecture^29^ and implemented using Python v3.7.12^42^ and PyTorch v1.8.0^43^. The U-Net takes five adjacent axial slices, stacked on the channel dimension, as input to produce predictions for only the center slice, using reflection padding to fill in the missing adjacent slices when necessary at the proximal and distal ends of the image. A 3D prediction is constructed by sweeping over the image axially, creating an independent prediction for each axial slice.

The double convolutional filters used in each layer of the U-Net are the same as in the original configuration with the modification of ‘same’-style padding in the convolutions and the addition of group normalization^44^ and dropout layers^45^. Finally, the U-Net does not directly produce a prediction of the cortical and trabecular segmentations as output. Instead, the outputs are of predicted level-set embedding fields^46^ for the endosteal and periosteal surfaces (see Fig. 1), where the sign of the field indicates whether a voxel is inside or outside of the embedded surface, and the magnitude of the field indicates the distance between a voxel and the embedded surface.

#### Loss functions

##### Classification accuracy

The primary loss function is based on the voxel-wise classification accuracy in the output slice. While the semantic segmentation of the image will consist of three non-overlapping binary masks (indicating trabecular, cortical, and background regions), the output of the network consists of two scalar fields: $\phi_{{{\text{endo}}}}$ and $\phi_{{{\text{peri}}}}$, the embedding fields for the endosteal and periosteal surfaces, respectively. The embedding fields are first converted to predicted probabilistic segmentations, $p_{{{\text{cort}}}}$, $p_{{{\text{trab}}}}$, and $p_{{{\text{back}}}}$, as follows:

3 $$\begin{array}{*{20}c} {p_{{{\text{trab}}}} = H_{\epsilon } \left( { - \phi_{{{\text{endo}}}} } \right), } \\ \end{array}$$

4 $$\begin{array}{*{20}c} {p_{{{\text{cort}}}} = H_{\epsilon } \left( {\phi_{{{\text{endo}}}} } \right)H_{\epsilon } \left( { - \phi_{{{\text{peri}}}} } \right), } \\ \end{array}$$

5 $$\begin{array}{*{20}c} {p_{{{\text{back}}}} = H_{\epsilon } \left( {\phi_{{{\text{peri}}}} } \right),} \\ \end{array}$$

where $H_{\epsilon } \left( \cdot \right)$ is an approximate and differentiable Heaviside function^47^ of the following form:

6 $$\begin{array}{*{20}c} {H_{\epsilon } \left( x \right) = \frac{1}{2} + \frac{1}{\pi }\tan^{ - 1} \left( {x/\epsilon } \right). } \\ \end{array}$$

The predicted segmentation probabilities are normalized to sum to one in each voxel:

7 $$\begin{array}{*{20}c} {p_{i}^{^{\prime}} = \frac{{p_{i} }}{{\mathop \sum \nolimits_{j} p_{j} }}, } \\ \end{array}$$

where $i$ and $j$ are indices over the three classes (background, cortical, trabecular). A cross-entropy loss function is used to compute the mean of the negative log-likelihood of the output, $L_{CE}$, across all voxels in the slice:

8 $$\begin{array}{*{20}c} {L_{CE} = \frac{1}{{\text{N}}}\mathop \sum \limits_{k} \mathop \sum \limits_{j} - s_{j} \log \left( {p_{j}^{^{\prime}} } \right), } \\ \end{array}$$

where $N$ is the number of voxels, $k$ is an index over all voxels, $j$ is an index over the classes, and $s_{j}$ are the corresponding binary values of the reference masks for each class at a voxel.

##### Curvature regularization

The curvature, $\kappa$, of a surface defined by an embedding in 2D can be calculated as the divergence of the surface normals^46^:

9 $$\begin{array}{*{20}c} {\kappa = \nabla \cdot \left( {\frac{\nabla \phi }{{\left| {\nabla \phi } \right|}}} \right), } \\ \end{array}$$

where $\nabla$ is the vector differential operator, $\nabla \phi$ is the gradient of the embedding field, and $\left| {\nabla \phi } \right|$ is the magnitude of the gradient of the embedding field. An accurate semantic segmentation of the cortical and trabecular compartments will have a characteristic smoothness, with the distribution of local curvatures falling within a specific band. Extreme curvatures in an output embedding field therefore would be non-physical and may indicate either overfitting or an incorrect model for the shape of the endosteal or periosteal surface. To penalize this, a regularizing loss function based on zero-level set local curvatures in embedding field $i$, $L_{\kappa ,i}$, is defined as:

10 $$\begin{array}{*{20}c} {L_{\kappa ,i} = \delta \left( {\phi_{i} } \right)ReLU\left( {\left( {\frac{{\kappa_{i} }}{{\kappa_{{{\text{thresh}}}} }}} \right)^{2} - 1} \right), } \\ \end{array}$$

where $\delta \left( {\phi_{i} } \right)$ is a binary mask that is activated where the embedding field crosses zero or is equal to zero, ${\text{ReLU}}\left( \cdot \right)$ is the rectified linear unit function^48^ and $\kappa_{{{\text{thresh}}}}$ is a curvature threshold below which the local value of $L_{\kappa ,i}$ will be zero. Based on preliminary investigation of surface curvatures in a subset of the *training* dataset, $\kappa_{{{\text{thresh}}}}$ was set at 0.005 μm^−1^ for both the endosteal and periosteal embedding fields.

##### Magnitude gradient regularization

When an embedding is a signed distance function, the magnitude of the gradient of the embedding field should be equal to one at all points not on the embedded surface^49^:

11 $$\begin{array}{*{20}c} {\left| {\nabla \phi } \right| = 1, where \phi \ne 0 } \\ \end{array}$$

This property motivates the construction of a second regularization loss function that penalizes the model for producing output embeddings that are not proper signed distance transforms of an embedded surface, formulated as such:

12 $$\begin{array}{*{20}c} {L_{{\left| {\nabla \phi } \right|,i}} = \left( {1 - \delta \left( \phi \right)} \right)\log^{2} \left| {\nabla \phi } \right|_{i} . } \\ \end{array}$$

The form of this loss function penalizes output embedding fields where the magnitude of the gradient is below or above one for voxels not directly on the embedded surface. There are two primary benefits: (1) extreme local variations in the output embedding fields could be indicative of overfitting, and (2) as the model outputs become more like a proper signed distance function, the curvature calculations for the loss function described in the preceding sub-section become more accurate.

##### Combined loss function

The combined loss function is a linear combination of the classification error, curvature regularization, and magnitude gradient regularization functions applied to each of the endosteal and periosteal embedding fields:

13 $$\begin{array}{*{20}c} {L_{{{\text{total}}}} = L_{CE} + \lambda_{\kappa } \left( {L_{{\kappa ,{\text{endo}}}} + L_{{\kappa ,{\text{peri}}}} } \right) + \lambda_{{\left| {\nabla \phi } \right|}} \left( {L_{{\left| {\nabla \phi } \right|,{\text{endo}}}} + L_{{\left| {\nabla \phi } \right|,{\text{peri}}}} } \right),} \\ \end{array}$$

where $\lambda_{\kappa }$ and $\lambda_{{\left| {\nabla \phi } \right|}}$ are regularization coefficients for the curvature and magnitude gradient regularization loss functions, respectively. Typically, optimal regularization coefficients would be determined using grid or random search in tandem with cross-validation^50^. However, this was infeasible due to computational resource constraints and so in this work each of these coefficients were set to $10^{ - 4}$. This value was selected empirically so that the classification accuracy-based loss would dominate at the start of training and that all losses would be of the same order of magnitude at convergence.

#### Training

The U-Net was trained on a research computing cluster node with two NVIDIA Tesla V100 GPUs for 25 epochs using the AdamW optimizer^51^ and the super-convergence one-cycle scheduling policy for optimizer hyper-parameters^52^. There were 10 epochs in a half-cycle and 5 convergence epochs, resulting in 25 total epochs and approximately 1.9 million iterations (25 epochs, 1822 images in the *training* set, and 42 batches per image). The minimum and maximum learning rates were 10^–4^ and 10^–3^, selected via learning rate range plot analysis, and the maximum and minimum momentums were 0.95 and 0.85. All other optimizer hyperparameters were left as default values. During each training epoch, the images in the *training* subset were shuffled then loaded sequentially. For each image, the axial slices were shuffled, then full slices were used sequentially for training with a batch size of 4. One inferential pass is performed on the full *validation* subset at the end of each training epoch.

### Morphological post-processing

Post-processing of output segmentations is necessary to ensure optimal morphometric analysis accuracy and precision. The specific post-processing approach used in this work is motivated by qualitative observations of the topology of the structures of interest: the cortical and trabecular compartments at the distal radius and tibia. First, there is only one simply connected region for each of the cortical and trabecular compartments. Second, there should be no background gaps between these compartments: there is only a single endosteal surface. Finally, the trabecular compartment should be fully separated from the background by a continuous cortical shell with a defined, and configurable, minimum physical thickness. Accordingly, a post-processing procedure was designed to ensure that these topological properties of the physiological structures are shared by the final predicted segmentations. In the following sub-sections, components of the post-processing procedure are described, followed by a high-level description of the overall procedure. The morphological image processing operations in the post-processing procedure were implemented using NumPy v1.16.6^53^, scikit-image v0.18.1^54^, and VTK^55^.

#### Iterative binary segmentation filter

The iterative binary segmentation filter is a modified version of the canonical alternating sequential filter^56^, in which open-close operations are applied to an image repeatedly with gradually increasing structural elements. A canonical ‘open’ is an erosion followed by a dilation, while a canonical ‘close’ is a dilation followed by an erosion. The first modification is to add a connectivity filter step to keep only the largest connected component of the foreground between the erosion and dilation of the open and keep only the largest connected component of the background between the dilation and erosion of the close. The second modification is to use different maximum structural element sizes for the open and close operations, which were radii of 5 voxels and 15 voxels, respectively. The purpose of this filter is to remove both union and subtractive noise and to ensure that the secondary bone (ulna, fibula) is not inadvertently included in the primary bone (radius, tibia) segmentation.

#### Minimum cortical shell filter

The minimum cortical shell filter takes a binary segmentation of the trabecular compartment as input and produces a binary segmentation of a cortical shell around this trabecular compartment as output. First, the trabecular binary segmentation is dilated by 8 voxels, or approximately 0.5 mm. Then the original trabecular segmentation is subtracted from the dilated segmentation, producing a cortical shell with a width of 8 voxels.

#### Morphological bone mask filter

The morphological bone mask filter is based on the first step of the gold-standard dual-thresholding algorithm^17^. The rescaled image is binarized using − 0.25 as a threshold in the normalized image intensity space, equivalent to a density-based threshold of 275 mg HA/cm^3^. A median filter is applied with a 3 × 3 × 1 (sagittal, coronal, axial) kernel, followed by the iterative binary segmentation filter (with maximum structural sizes of radius 3 voxels and radius 15 voxels for the open and close operations, respectively). The result is a purely morphologically derived binary image containing a rough mask of the entire primary bone. The purpose of generating this mask and combining it with the bone mask generated by the U-Net is to reduce the likelihood of a catastrophic error—i.e., the bone mask missing a portion of the primary bone—at the cost of potentially reducing the accuracy of the periosteal contour.

#### Post-processing procedure

**Figure 5:**
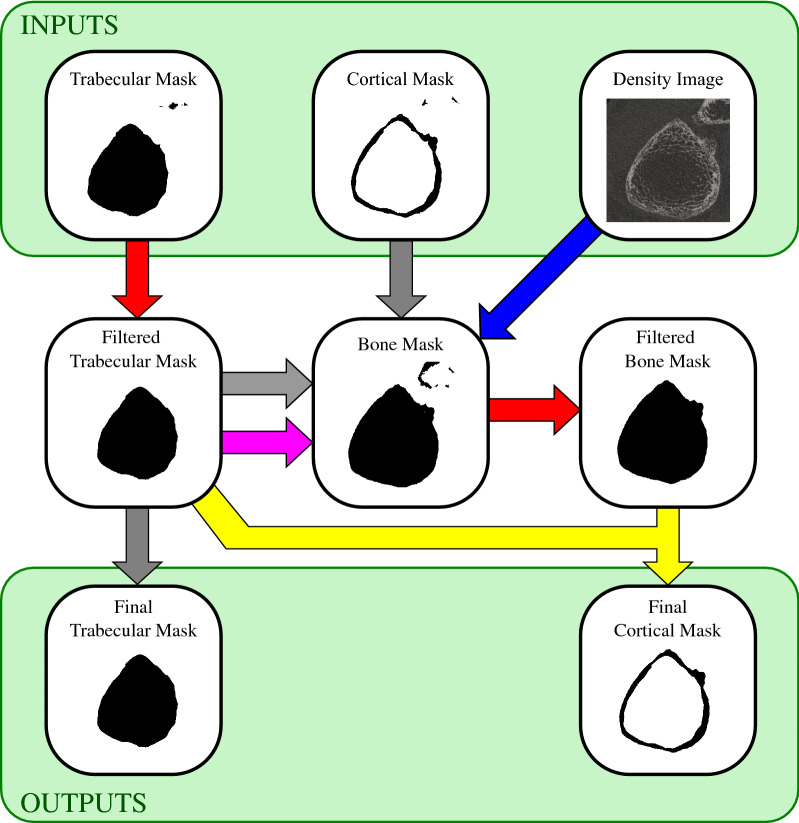
Schematic of post-processing procedure visualised using a distal tibia image. The top row shows the inputs while the bottom row shows the outputs. Block arrows correspond to composite morphological filtering operations: grey—the mask is simply copied, red—iterative binary segmentation filter, pink—minimum cortical shell filter, blue—morphological bone mask filter, and yellow—subtraction (the filtered trabecular mask is subtracted from the filtered bone mask). When multiple arrows converge on the same output mask, this indicates the outputs were combined (union).

The U-Net-output embeddings for the endosteal and periosteal surfaces are converted to two binary images, or masks: the cortical mask is defined as the region between the endosteal and periosteal surfaces ($\phi_{{{\text{endo}}}} > 0$ and $\phi_{{{\text{peri}}}} < 0$), and the trabecular mask is defined as the region inside of the endosteal surface ($\phi_{{{\text{endo}}}} < 0$). This conversion procedure is like what is done during training for calculation of the classification accuracy, except here binary segmentations are computed by checking if the embedding fields are greater than or less than zero, rather than using differentiable approximations to generate fuzzy segmentations. Figure 5 shows a flow-chart of the complete morphological post-processing procedure that follows, along with incremental visualizations of the outputs of the major steps and the corresponding operations explained in detail in the figure caption. The final post-processing outputs are the filtered cortical and trabecular masks. During inference or testing, these can be automatically saved in a manufacturer-specific image format for further processing, such as morphometric analysis.

### Evaluation and metrics

After the U-Net was trained using the *training* subset and the post-processing procedure was developed using the *validation* subset, the parameters of the U-Net and post-processing procedure were frozen and used in sequence to obtain predicted segmentations on the *test* and *precision* subsets. These predicted segmentations were compared to the corresponding reference segmentations to evaluate the accuracy and reproducibility of the proposed segmentation algorithm. All statistical analysis was performed using Python v3.7.12^42^, statsmodels v0.11.1^57^, and SciPy v.1.6.1^58^ with a significance threshold of 5% for all statistical testing.

Predicted and reference segmentations were compared on the *test* subset both by using standard segmentation quality metrics and by comparing the quantitative outputs of the standard HR-pQCT extended cortical analysis using the standard manufacturer-provided procedure^13,59^. The standard segmentation quality metrics used were the Dice similarity coefficient (DSC), the Jaccard similarity coefficient (JSC), and the average and maximum of the symmetric surface distances (SSD)^60^, hereafter referred to as ASSD and Hausdorff distance, respectively (implemented using SimpleITK v2.0.2^61^). The quantitative analysis outputs used for comparison were: total bone mineral density (Tt.BMD) and area (Tt.Ar), cortical bone mineral density (Ct.BMD), thickness (Ct.Th), porosity (Ct.Po), and area (Ct.Ar), and trabecular volumetric bone mineral density (Tb.BMD), bone volume fraction (Tb.BV/TV), number (Tb.N), thickness (Tb.Th), separation (Tb.Sp), and area (Tb.Ar)^13^. For each parameter at each scan site, the paired prediction and reference outputs were compared using both direct linear correlation analysis and by the difference and mean values in Bland–Altman (or Tukey mean difference) plots.

To investigate the performance of the proposed protocol in particularly adverse situations where a human operator would find the most difficulty in correcting the endosteal contour, two sub-groups were also analyzed separately for each scan site: The “low cortical thickness” sub-groups, which were composed of the radius and tibia images in the bottom quartile for Ct.Th for each scan site, and the “high cortical porosity” sub-groups, which were composed of the radius and tibia images in the top quartile for Ct.Po for each scan site.

Finally, the precision of the proposed algorithm was compared to the gold-standard protocol by calculating the least significant change (LSC) and the root-mean-squared percentage coefficient of variation (RMS %CV) on the pairs of same-day repeat scans in the *precision* subset^41^. The pairs of repeat-scan images were registered, and the common volume shared between the two was computed. Then, morphometric analysis was applied only to the common volume using the predicted and reference masks for each image^62^. The LSC and RMS %CV were calculated for each of the standard quantitative analysis outputs. Normality of the individual standard deviations from the paired *precision* data were assessed separately for the predicted and reference analysis outputs using D’Agostino and Pearson’s method^63^, and the significance of the differences in *precision* outputs were assessed using independent Wilcoxon signed-rank tests^64^. As with the *test* subset, this analysis procedure was then repeated for “low cortical thickness” and “high cortical porosity” sub-groups formed separately for each scan site.

### Visualizations

Plots were generated using pandas v1.2.4^65^, Matplotlib v3.4.3^66^, and seaborn v0.11.2^67^. Volumetric renderings were created using vtkbone (https://github.com/Numerics88/vtkbone), VTK v8.2.0^55^, and PyVista v0.33.2^68^.

## Conclusion

We have presented and validated a novel, fully automated algorithm for the semantic segmentation of HR-pQCT distal radius and tibia images. The proposed algorithm requires no human input or oversight and is faster, as accurate, and as precise or more precise when compared to the current gold-standard semi-automated approach. In its current form, it can be seamlessly integrated into standard workflows for HR-pQCT morphometric analysis with radius and tibia images. Future work will focus on translating this approach to additional scan sites.

## Supplementary Information

Supplementary Information.

## Data Availability

The datasets used in the current study are not publicly available to protect confidentiality and privacy of participants. They can be made available to other researchers for non-commercial use, upon reasonable request, from Steven K. Boyd at skboyd@ucalgary.ca.

## Code availability

All code used for model development, training, inference, post-processing, visualization, and statistical analysis is publicly available in the following GitHub repository: https://github.com/Bonelab/HR-pQCT-Segmentation. The trained model weights used for segmentation of the *test* and *precision* data in this work are available upon reasonable request.
