# Supplementary material for: Automatic segmentation of trabecular and cortical compartments in HR-pQCT images using an embedding-predicting U-Net and morphological post-processing
Source: Sci Rep. 2023 Jan 5;13:252. doi: 10.1038/s41598-022-27350-0 (PMC9816121; doi:10.1038/s41598-022-27350-0)
Supplement: Supplementary file 1 — Supplementary Information. [file 41598_2022_27350_MOESM1_ESM.pdf]

Table S.1: Results of linear regression and Bland-Altman analysis on the samples in the held-out *test* dataset with reference cortical thickness in the bottom 25% for each scan site ("low cortical thickness"), comparing the predicted morphometric outputs, obtained using predicted segmentations, and the reference morphometric outputs, obtained using reference segmentations.

		Bland-Altman	Linear Regression		
		Mean Error (95% LOA[a])	Slope (95% C.I.[b])	Intercept (95% C.I.[b])	R^2
**Radius (n=46)**					
Tt.BMD	mg HA/cm^3	1.0 (-0.2, 2.2)	0.990 (0.987,0.993)	3.4 (2.6, 4.2)	>0.999
Ct.BMD	mg HA/cm^3	-8.6 (-31.4, 14.2)	1.010 (0.956,1.065)	-17 (-63, 29)	0.968
Tb.BMD	mg HA/cm^3	-0.2 (-3.7, 3.3)	0.986 (0.974,0.998)	1.5 (-0.1, 3.1)	0.998
Ct.Th	mm	0.00 (-0.05, 0.04)	0.921 (0.850,0.991)	0.060 (0.004, 0.116)	0.937
Ct.Po	%	-0.03 (-0.30, 0.23)	0.887 (0.835,0.939)	0.054 (0.001, 0.106)	0.962
Tb.BV/TV	%	-0.030 (-0.522, 0.462)	0.984 (0.971,0.997)	0.248 (0.008, 0.488)	0.998
Tb.N	mm^{-1}	0.000 (-0.002, 0.002)	1.001 (1.000,1.002)	-0.001 (-0.003, 0.000)	>0.999
Tb.Th	mm	-0.000 (-0.006, 0.005)	0.909 (0.845,0.973)	0.020 (0.006, 0.034)	0.946
Tb.Sp	mm	-0.000 (-0.003, 0.002)	0.999 (0.998,1.000)	0.000 (-0.000, 0.001)	>0.999
Tt.Ar	mm^2	1.1 (-0.3, 2.4)	1.000 (0.996,1.005)	0.97 (-0.29, 2.22)	>0.999
Ct.Ar	mm^2	0.9 (-1.9, 3.7)	0.955 (0.895,1.016)	2.99 (0.13, 5.84)	0.956
Tb.Ar	mm^2	0.1 (-2.4, 2.7)	0.993 (0.984,1.002)	1.77 (-0.35, 3.88)	0.999
**Tibia (n=50)**					
Tt.BMD	mg HA/cm^3	0.1 (-0.2, 0.5)	0.998 (0.997,0.999)	0.6 (0.3, 0.9)	>0.999
Ct.BMD	mgHA/cm^3	0.3 (-19.6, 20.3)	0.989 (0.960,1.018)	9.3 (-14.5, 33.0)	0.989
Tb.BMD	mgHA/cm^3	0.4 (-3.4, 2.6)	0.991 (0.979,1.003)	0.9 (-0.8, 2.6)	0.998
Ct.Th	mm	0.002 (-0.069, 0.073)	0.927 (0.856,0.998)	0.083 (0.003, 0.163)	0.931
Ct.Po	%	-0.025 (-0.627, 0.576)	0.984 (0.940,1.028)	0.025 (-0.139, 0.189)	0.976
Tb.BV/TV	%	-0.054 (-0.455, 0.347)	0.989 (0.977,1.000)	0.186 (-0.059, 0.432)	0.998
Tb.N	mm^{-1}	-0.000 (-0.004, 0.004)	0.999 (0.997,1.002)	0.001 (-0.002, 0.004)	>0.999
Tb.Th	mm	-0.001 (-0.006, 0.004)	0.917 (0.884,0.951)	0.019 (0.011, 0.027)	0.984
Tb.Sp	mm	-0.000 (-0.002, 0.002)	0.999 (0.997,1.000)	0.001 (-0.000, 0.002)	>0.999
Tt.Ar	mm^2	0.4 (-0.4, 1.3)	1.000 (0.999,1.001)	0.26 (-0.53, 1.06)	>0.999
Ct.Ar	mm^2	0.3 (-5.9, 6.4)	0.901 (0.849,0.953)	10.16 (4.89, 15.43)	0.960
Tb.Ar	mm^2	0.1 (-5.9, 6.1)	1.007 (0.999,1.015)	-4.56 (-9.64, 0.52)	0.999

[a] 95% limits of agreement (LOA) are the mean error plus or minus 1.96 times the standard deviation of the errors.

[b] 95% confidence interval (C.I.) are the estimated slope or intercept plus or minus 1.96 times the estimated standard error of the estimate, as reported by statsmodels' ordinary least squares (OLS) linear regressor after being fit to the data.

Table S.2: Results of linear regression and Bland-Altman analysis on the samples in the held-out *test* dataset with reference cortical porosity in the top 25% for each scan site ("high cortical porosity"), comparing the predicted morphometric outputs, obtained using predicted segmentations, and the reference morphometric outputs, obtained using reference segmentations.

		Bland-Altman	Linear Regression		
		Mean Error (95% LOA[a])	Slope (95% C.I.[b])	Intercept (95% C.I.[b])	R^2
**Radius (n=46)**					
Tt.BMD	mg HA/cm^3	0.5 (-0.6, 1.6)	0.995 (0.993, 0.997)	2.1 (1.5, 2.7)	>0.999
Ct.BMD	mg HA/cm^3	3.5 (-23.4, 30.5)	0.982 (0.919, 1.045)	18.8 (-34.1, 71.7)	0.955
Tb.BMD	mg HA/cm^3	1.0 (-4.7, 6.8)	0.994 (0.975, 1.013)	2.1 (-1.1, 5.3)	0.996
Ct.Th	mm	-0.025 (-0.091, 0.041)	0.946 (0.901, 0.992)	0.033 (-0.017, 0.083)	0.974
Ct.Po	%	-0.217 (-0.725, 0.291)	0.813 (0.696, 0.929)	0.116 (-0.102, 0.333)	0.810
Tb.BV/TV	%	0.165 (-0.620, 0.950)	0.994 (0.975, 1.012)	0.308 (-0.126, 0.741)	0.996
Tb.N	mm^{-1}	0.001 (-0.004, 0.006)	1.000 (0.997, 1.002)	0.001 (-0.003, 0.005)	>0.999
Tb.Th	mm	0.001 (-0.008, 0.010)	0.968 (0.897, 1.039)	0.008 (-0.008, 0.025)	0.942
Tb.Sp	mm	-0.001 (-0.005, 0.003)	0.997 (0.995, 0.999)	0.001 (-0.001, 0.002)	>0.999
Tt.Ar	mm^2	0.7 (-0.4, 1.8)	1.000 (0.998, 1.002)	0.72 (0.07, 1.36)	>0.999
Ct.Ar	mm^2	-0.8 (-5.4, 3.8)	0.949 (0.917, 0.981)	2.59 (0.39, 4.79)	0.987
Tb.Ar	mm^2	1.4 (-3.2, 6.1)	1.009 (0.999, 1.020)	-0.72 (-3.19, 1.740)	0.999
**Tibia (n=50)**					
Tt.BMD	mg HA/cm^3	0.0 (-0.2, 0.3)	0.999 (0.998, 1.000)	0.3 (0.0, 0.5)	>0.999
Ct.BMD	mgHA/cm^3	7.2 (-18.8, 33.3)	0.935 (0.883, 0.986)	56.0 (17.3, 94.7)	0.963
Tb.BMD	mgHA/cm^3	1.4 (-4.0, 6.9)	0.983 (0.964, 1.002)	4.1 (1.0, 7.2)	0.995
Ct.Th	mm	-0.034 (-0.145, 0.077)	0.916 (0.879, 0.954)	0.088 (0.032, 0.144)	0.979
Ct.Po	%	-0.217 (-1.156, 0.723)	0.893 (0.791, 0.994)	0.382 (-0.199, 0.963)	0.861
Tb.BV/TV	%	0.204 (-0.521, 0.930)	0.981 (0.963, 1.000)	0.641 (0.192, 1.091)	0.995
Tb.N	mm^{-1}	0.001 (-0.006, 0.009)	0.997 (0.992, 1.001)	0.006 (0.000, 0.011)	>0.999
Tb.Th	mm	0.001 (-0.007, 0.010)	0.982 (0.931, 1.033)	0.006 (-0.007, 0.019)	0.968
Tb.Sp	mm	-0.001 (-0.006, 0.003)	0.997 (0.994, 1.000)	0.001 (-0.002, 0.003)	>0.999
Tt.Ar	mm^2	0.3 (-0.3, 0.9)	1.000 (1.000, 1.001)	0.02 (-0.44, 0.48)	>0.999
Ct.Ar	mm^2	-3.0 (-13.5, 7.5)	0.947 (0.907, 0.986)	3.98 (-1.40, 9.35)	0.978
Tb.Ar	mm^2	3.2 (-7.2, 13.6)	0.992 (0.981, 1.004)	7.86 (0.91, 14.81)	0.998

[a] 95% limits of agreement (LOA) are the mean error plus or minus 1.96 times the standard deviation of the errors.

[b] 95% confidence interval (C.I.) are the estimated slope or intercept plus or minus 1.96 times the estimated standard error of the estimate, as reported by statsmodels' ordinary least squares (OLS) linear regressor after being fit to the data.

Table S.3: Root-mean-square standard deviations (RMS SD) compared between the proposed algorithm (U-Net) and the current semi-automated gold standard (Standard) segmentation protocols on the held-out *precision* dataset.

		RMS SD	
		U-Net[a]	Standard[b]
**Radius (n=71)**			
Tt.BMD	mg HA/cm^3	0.91	2.01
Ct.BMD	mg HA/cm^3	2.41	6.90
Tb.BMD	mg HA/cm^3	0.68	0.85
Ct.Th	mm	0.0081	0.0092
Ct.Po	%	0.081	0.089
Tb.BV/TV	%	0.18	0.20
Tb.N	mm^{-1}	0.021	0.021
Tb.Th	mm	0.0015	0.0017
Tb.Sp	mm	0.0078	0.0080
Tt.Ar	mm^2	2.24	1.81
Ct.Ar	mm^2	0.53	0.64
Tb.Ar	mm^2	1.93	2.27
**Tibia (n=85)**			
Tt.BMD	mg HA/cm^3	1.34	1.35
Ct.BMD	mg HA/cm^3	3.02	3.78
Tb.BMD	mg HA/cm^3	1.01	1.11
Ct.Th	mm	0.0094	0.0115
Ct.Po	%	0.27	0.30
Tb.BV/TV	%	0.18	0.19
Tb.N	mm^{-1}	0.041	0.041
Tb.Th	mm	0.0015	0.0018
Tb.Sp	mm	0.016	0.016
Tt.Ar	mm^2	2.03	0.99
Ct.Ar	mm^2	0.51	0.87
Tb.Ar	mm^2	1.78	0.97

[a] U-Net: automated segmentation algorithm using a U-Net and morphological post-processing.
[b] Standard: Current standard semi-automated segmentation protocol.

Table S.4: Root-mean-square percentage coefficient of variation (RMS %CV) compared between the proposed algorithm (U-Net) and the current semi-automated gold standard (Standard) segmentation protocols on samples from the held-out *precision* dataset in the top quartile for Ct.Po (high porosity) and in the bottom quartile for Ct.Th (low thickness). Quartiles were extracted separately for each scan site.

		RMS %CV (low thickness)		Wilcoxon	RMS %CV (high porosity)		Wilcoxon
		U-Net[a]	Standard[b]	*p* Value	U-Net[a]	Standard[b]	*p* Value
**Radius (n=18, 18)**							
Tt.BMD	mg HA/cm^3	0.29	0.92	0.550	0.29	0.30	0.740
Ct.BMD	mg HA/cm^3	0.26	1.13	0.442	0.19	0.32	0.012[c]
Tb.BMD	mg HA/cm^3	0.63	0.77	0.072	0.51	0.65	0.463
Ct.Th	mm	0.54	0.97	0.016[c]	0.92	0.95	0.347
Ct.Po	%	9.14	14.32	0.018[c]	7.51	8.88	0.072
Tb.BV/TV	%	1.43	1.52	1.000	0.86	0.89	0.966
Tb.N	mm^{-1}	0.92	0.98	0.003[c]	1.45	1.47	0.442
Tb.Th	mm	0.64	0.64	0.468	0.65	0.93	0.671
Tb.Sp	mm	1.00	1.07	0.181	0.97	0.99	0.369
Tt.Ar	mm^2	0.95	1.06	0.523	0.74	0.34	0.495
Ct.Ar	mm^2	1.11	1.74	1.000	0.62	0.75	0.932
Tb.Ar	mm^2	0.95	1.46	0.119	0.94	0.58	0.181
**Tibia (n=21, 21)**							
Tt.BMD	mg HA/cm^3	0.70	0.72	0.301	0.69	0.71	0.756
Ct.BMD	mg HA/cm^3	0.43	0.48	0.147	0.44	0.52	0.216
Tb.BMD	mg HA/cm^3	0.86	1.02	0.472	0.82	0.93	0.136
Ct.Th	mm	0.74	0.89	0.973	0.66	0.90	0.865
Ct.Po	%	9.18	10.36	0.575	9.91	9.05	0.683
Tb.BV/TV	%	0.99	0.97	0.955	1.06	0.99	0.145
Tb.N	mm^{-1}	1.80	1.81	0.019[c]	3.10	3.11	0.002[c]
Tb.Th	mm	0.50	0.66	0.432	0.52	0.69	0.191
Tb.Sp	mm	1.13	1.15	0.452	2.10	2.10	0.320
Tt.Ar	mm^2	0.20	0.09	0.006[d]	0.31	0.17	0.179
Ct.Ar	mm^2	0.45	0.87	0.014[c]	0.41	0.76	<0.001[c]
Tb.Ar	mm^2	0.24	0.14	0.973	0.33	0.17	0.633

[a] U-Net: automated segmentation algorithm using a U-Net and morphological post-processing.
[b] Standard: Current standard semi-automated segmentation protocol.
[c] Wilcoxon signed-rank test indicates significantly lower individual standard deviations with automated segmentation algorithm as compared to the standard semi-automated protocol.
[d] Wilcoxon signed-rank test indicates significantly higher individual standard deviations with automated segmentation algorithm as compared to the standard semi-automated protocol.

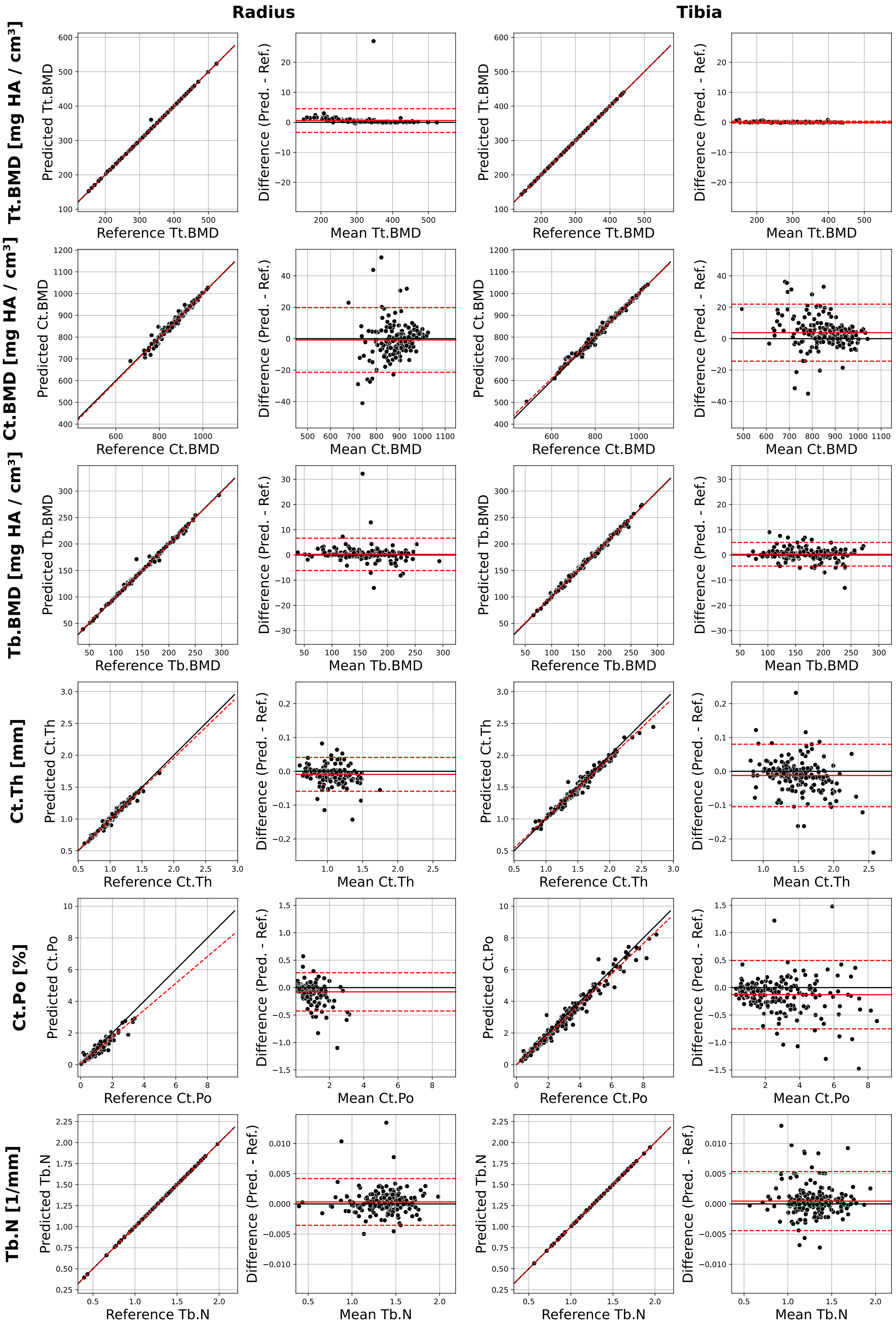

Figure S.1: Linear regression and Bland-Altman plots comparing results of morphometric analysis using predicted and reference masks for Tt.BMD, Ct.BMD, Tb.BMD, Ct.Th, Ct.Po, and Tb.N.

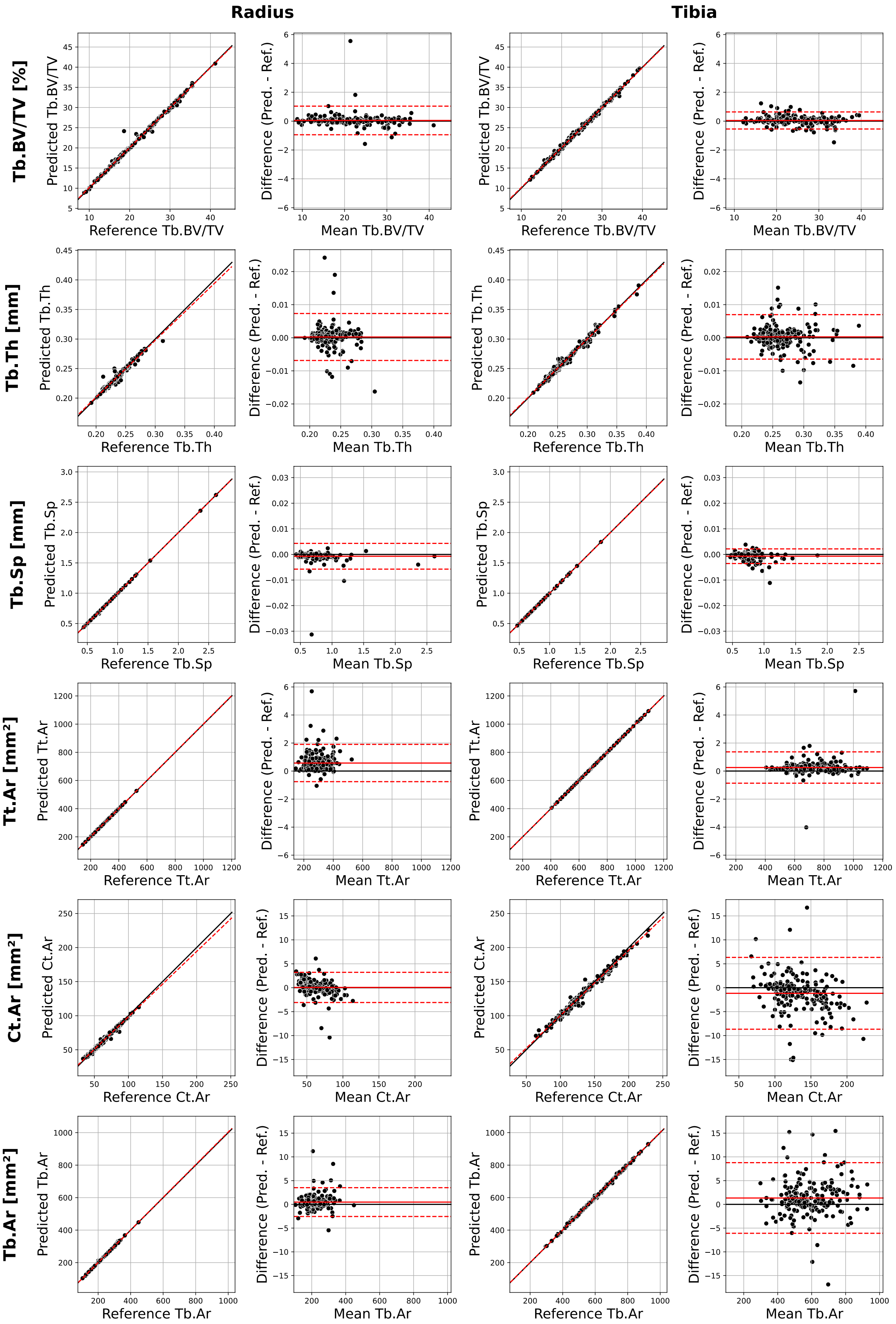

Figure S.2: Linear regression and Bland-Altman plots comparing results of morphometric analysis using predicted and reference masks for Tb.BV/TV, Tb.Th, Tb.Sp, Tt.Ar, Ct.Ar, and Tb.Ar.

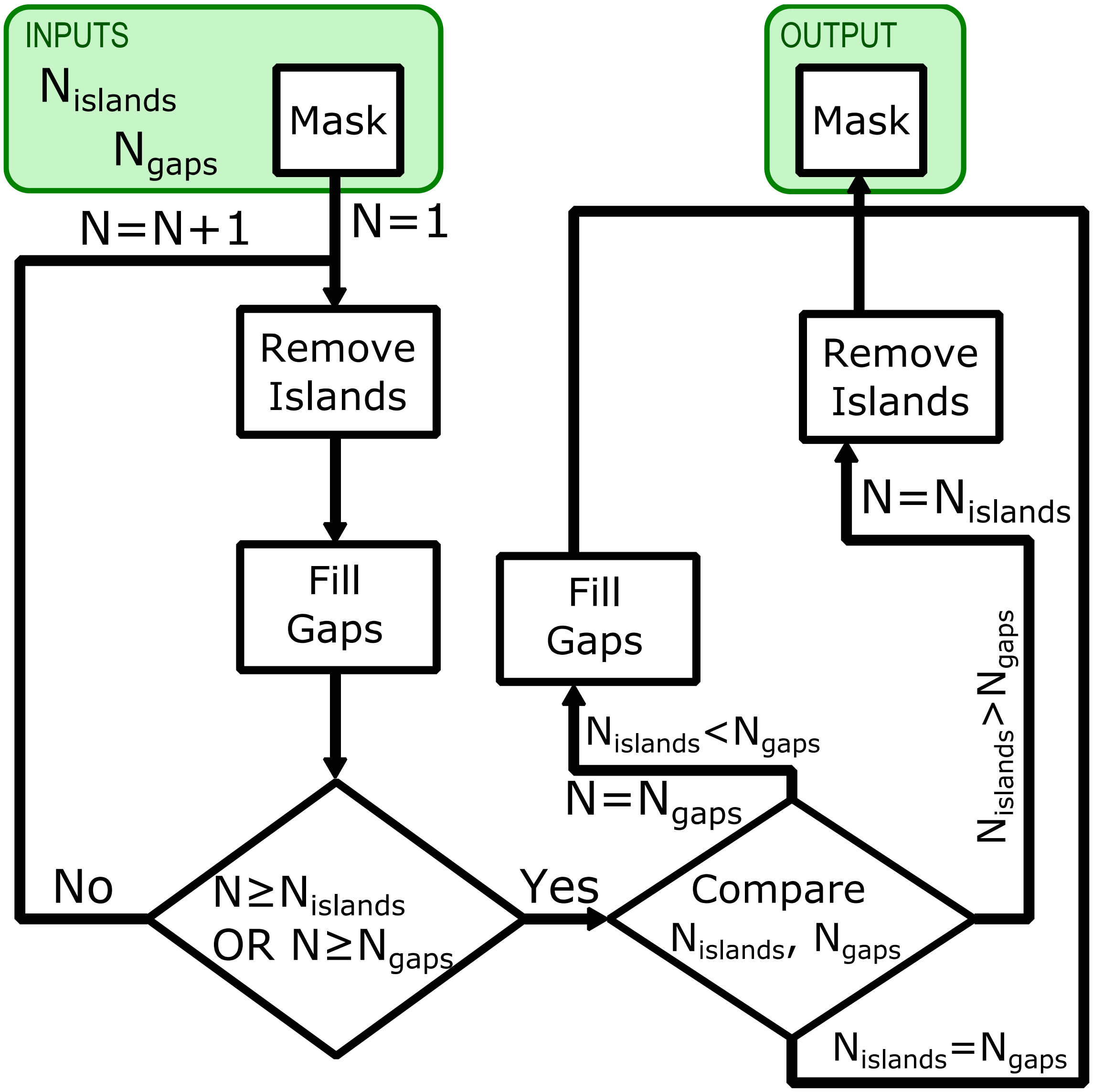

Figure S.3: Schematic of the iterative binary segmentation filter, which is based on an alternating sequential filter with connected components filtering interposed between dilations and erosions. ‘Remove Islands’ refers to performing N 3x3x3 erosions, connected component filtering to keep only the largest connected foreground region, and N 3x3x3 dilations. ‘Fill Gaps’ refers to performing N 3x3x3 dilations, connected component filtering to keep only the largest connected background region, and N 3x3x3 erosions. These two operations alternate with iteratively increasing N until N exceeds $N_{islands}$ or N_{gaps}. At this point, if either of $N_{islands}$ or N_{gaps} exceed the other the corresponding operation is applied to the binary mask one final time with the corresponding number of erosions and dilations.
